# Haematuria cancer risk score as a predictor of muscle‐invasive bladder cancer

**DOI:** 10.1002/bco2.70125

**Published:** 2025-12-03

**Authors:** Serdar Turan, Cem Tugrul Gezmis, Nusret Can Cilesiz, Mehmet Uzut, Mustafa Satılmısoglu, Rifat Burak Ergul, Mustafa Bahadır Can Balcı

**Affiliations:** ^1^ Department of Urology Taksim Training and Research Hospital İstanbul Türkiye; ^2^ Department of Urology, Istanbul Faculty of Medicine Istanbul University Istanbul Türkiye

**Keywords:** bladder cancer, Haematuria Cancer Risk Score, muscle‐invasive bladder cancer, prognostic factors, risk stratification

## Abstract

**Objective:**

To evaluate the prognostic performance of the Haematuria Cancer Risk Score (HCRS) for predicting muscle‐invasive bladder cancer (MIBC) in patients presenting with haematuria.

**Methods:**

This retrospective study analysed newly diagnosed urothelial carcinoma cases identified during haematuria evaluations between 2018 and 2023. Pathological staging was based on the highest grade or stage from TUR or re‐TUR specimens. HCRS was calculated using age, sex, haematuria type, and smoking status. The primary outcome was muscle‐invasive disease (≥T2). Associations were examined using univariate logistic regression, and discriminatory performance was evaluated with ROC analysis and bootstrap‐derived 95% confidence intervals.

**Results:**

Among 162 patients, 45 (27.8%) had MIBC. The median HCRS was higher in patients with MIBC than in those with non‐muscle‐invasive disease (6.49 [6.08–6.96] vs. 6.16 [5.44–6.94]; *p* = 0.04). Univariate analysis showed a significant association between HCRS and MIBC (odds ratio = 1.78; 95% CI 1.03–3.06; *p* = 0.039). ROC analysis demonstrated limited discriminative performance (AUC = 0.60; 95% CI 0.53–0.67). At the Youden cut‐off of 6.04, sensitivity was 77.8% and specificity 48.3%. An integer threshold of HCRS ≥6 yielded comparable performance, supporting clinical applicability.

**Conclusions:**

Higher HCRS values were modestly associated with muscle invasion, although predictive performance was limited. The HCRS may support risk stratification in haematuria pathways by flagging patients at higher risk of muscle invasion—particularly when interpreted together with cystoscopy and imaging findings.

## 1 INTRODUCTION

Haematuria is one of the most common urological symptoms and presents a diagnostic challenge because its causes range from benign conditions to urothelial carcinoma.^1^, ^2^ Although most cases are ultimately non‐malignant, the need to exclude cancer necessitates extensive diagnostic evaluation, including cystoscopy and imaging, which increases cost and patient burden.^3^, ^4^ Risk‐based triage systems are therefore essential to optimize evaluation strategies while maintaining diagnostic safety.^5^

To address this need, Tan et al. developed the Haematuria Cancer Risk Score (HCRS), a pragmatic tool that combines age, sex, haematuria type (gross or microscopic) and smoking status to estimate the likelihood of bladder cancer.^6^ The score demonstrated good discrimination (AUC 0.835; 95% CI: 0.789–0.880) and satisfactory calibration, with no significant overfitting, and has been externally validated in diverse populations.

Integration of the HCRS into ultrasonography‐based or ‘imaging‐first’ diagnostic pathways has been proposed to safely reduce unnecessary cystoscopies without compromising detection rates.^5^, ^7^ Despite its strong diagnostic performance, the prognostic potential of the HCRS remains underexplored. Because its constituent variables—visible haematuria, smoking, older age and male sex—are also associated with advanced disease and aggressive tumour biology, higher HCRS values may plausibly indicate an increased risk of muscle invasion.^8^, ^9^ Determining whether this simple, non‐invasive score can identify patients with biologically aggressive disease would extend its clinical utility beyond diagnostic triage towards risk stratification and surveillance planning.

The present study aimed to evaluate the association between HCRS and muscle‐invasive bladder cancer (MIBC) in a retrospective cohort of 162 patients with histologically confirmed urothelial carcinoma. We hypothesized that higher HCRS values would be associated with an increased probability of muscle invasion. Unlike prior analyses, multivariable adjustment for the score's components was intentionally avoided to prevent collinearity, focusing instead on its overall discriminative performance and clinical applicability. Ultimately, the study sought to clarify whether the HCRS could support risk stratification in haematuria pathways by flagging patients at higher risk of muscle invasion, particularly when combined with cystoscopy and imaging findings.

## 2 MATERIALS AND METHODS

### 2.1 Study design and population

This retrospective study included 162 consecutive patients who underwent diagnostic evaluation for haematuria at our tertiary referral centre between January 2018 and December 2023. Patients with histopathologically confirmed urothelial carcinoma of the bladder were identified from institutional records. Individuals with incomplete data for tumour stage, haematuria type or HCRS components were excluded. Only patients with a new diagnosis of bladder cancer were included. If muscle tissue sampling was not available in the initial transurethral resection (TUR) specimen, or if the re‐TUR specimen demonstrated a higher pathological grade or stage, the higher value was recorded for analysis.

Patients with non‐urothelial malignancy and primary CIS detected in their final pathology were excluded from the study. The study was approved by the Institutional Ethics Committee of Taksim Training and Research Hospital and conducted in accordance with the Declaration of Helsinki; informed consent was waived due to the retrospective design.

### 2.2 HCRS

For each patient, the HCRS was calculated according to the original scoring system by Tan et al., which integrates age, sex, haematuria type (gross vs. microscopic) and smoking status.

Haematuria cancer risk score=0.055×age+1.348×haematuria type+0.576×gender+0.413×ex−smoker+0.943×current−smoker

$$\begin{array}{l} \left(0=\text{female, }1=\text{male}\right),\\ \text{type of haematuria}\left(0=\text{non}-\text{visible haematuria, }1=\mathrm{VH}\right),\\ \text{smoking history}(0=\text{non}-\text{smoker, }1=\mathrm{ex}-\text{smoker, }2\\ =\text{current smoker, }3=\text{missing}) \end{array}$$

### 2.3 Outcomes and variables

The primary outcome was MIBC, defined as pathological stage ≥T2. Exploratory variables included carcinoma in situ (CIS) and variant histology.

### 2.4 Statistical analysis

All analyses were conducted in R (v4.3.2; R Foundation for Statistical Computing, Vienna, Austria). Continuous variables were assessed for normality using the Shapiro–Wilk test and expressed as mean ± SD or median (IQR), as appropriate. Categorical variables were presented as *n* (%) and compared using the Wilcoxon rank‐sum or chi‐square/Fisher's exact tests. The association between HCRS and MIBC was evaluated using univariate logistic regression, and results were expressed as odds ratios (ORs) with 95% confidence intervals (CIs). Because HCRS is a composite of age, sex, haematuria type and smoking, multivariable models including these components were not fitted to avoid collinearity. Discriminative performance was assessed with receiver operating characteristic (ROC) analysis using the pROC package. The area under the curve (AUC) was reported with bootstrap 95% CIs (2000 iterations). The Youden index determined the optimal cut‐off, and corresponding sensitivity, specificity, positive predictive value (PPV) and negative predictive value (NPV) were calculated. A two‐sided *p* value <0.05 was considered statistically significant.

## 3 RESULTS

### 3.1 Study population

One hundred sixty‐two patients with pathologically confirmed urothelial carcinoma who underwent diagnostic evaluation for haematuria between 2018 and 2023 were included. The median age was 69 years (interquartile range [IQR] 63–76), and 82.7% were male. Visible haematuria was present in 75.9% of the cohort, and 45 patients (27.8%) had MIBC (≥T2). Variant histology was observed in 1.9%, and CIS in 4.9% of patients (Table 1).

**TABLE 1:** Baseline characteristics of the study cohort (*N* = 162).

Characteristic	Overall (*n* = 162)	Non‐MIBC (*n* = 117)	MIBC (*n* = 45)	*p* value
Age, median [IQR], years	69 [63–76]	68 [62–75]	71 [66–78]	0.06
Sex, *n* (%)				0.12
Male	134 (82.7%)	94 (80.3%)	40 (88.9%)	
Female	28 (17.3%)	23 (19.7%)	5 (11.1%)	
Haematuria type, *n* (%)				**0.03**
Gross (visible)	123 (75.9%)	84 (71.8%)	39 (86.7%)	
Microscopic	39 (24.1%)	33 (28.2%)	6 (13.3%)	
Smoking status, *n* (%)				0.09
Never	52 (32.1%)	41 (35.0%)	11 (24.4%)	
Former	68 (42.0%)	48 (41.0%)	20 (44.4%)	
Current	42 (25.9%)	28 (23.9%)	14 (31.1%)	
HCRS, median [IQR]	6.28 [5.62–6.95]	6.16 [5.44–6.94]	6.49 [6.08–6.96]	**0.04**
Variant histology, *n* (%)	3 (1.9%)	2 (1.7%)	1 (2.2%)	0.80
CIS, *n* (%)	8 (4.9%)	6 (5.1%)	2 (4.4%)	0.85

*Notes*: Data are presented as median (IQR) or *n* (%). *p* values were derived using Wilcoxon rank‐sum (continuous) or chi‐square/Fisher's exact test (categorical). Bold values indicate statistical significance (*p* < 0.05).

### 3.2 Comparison of HCRS between tumour stages

**FIGURE 1:**
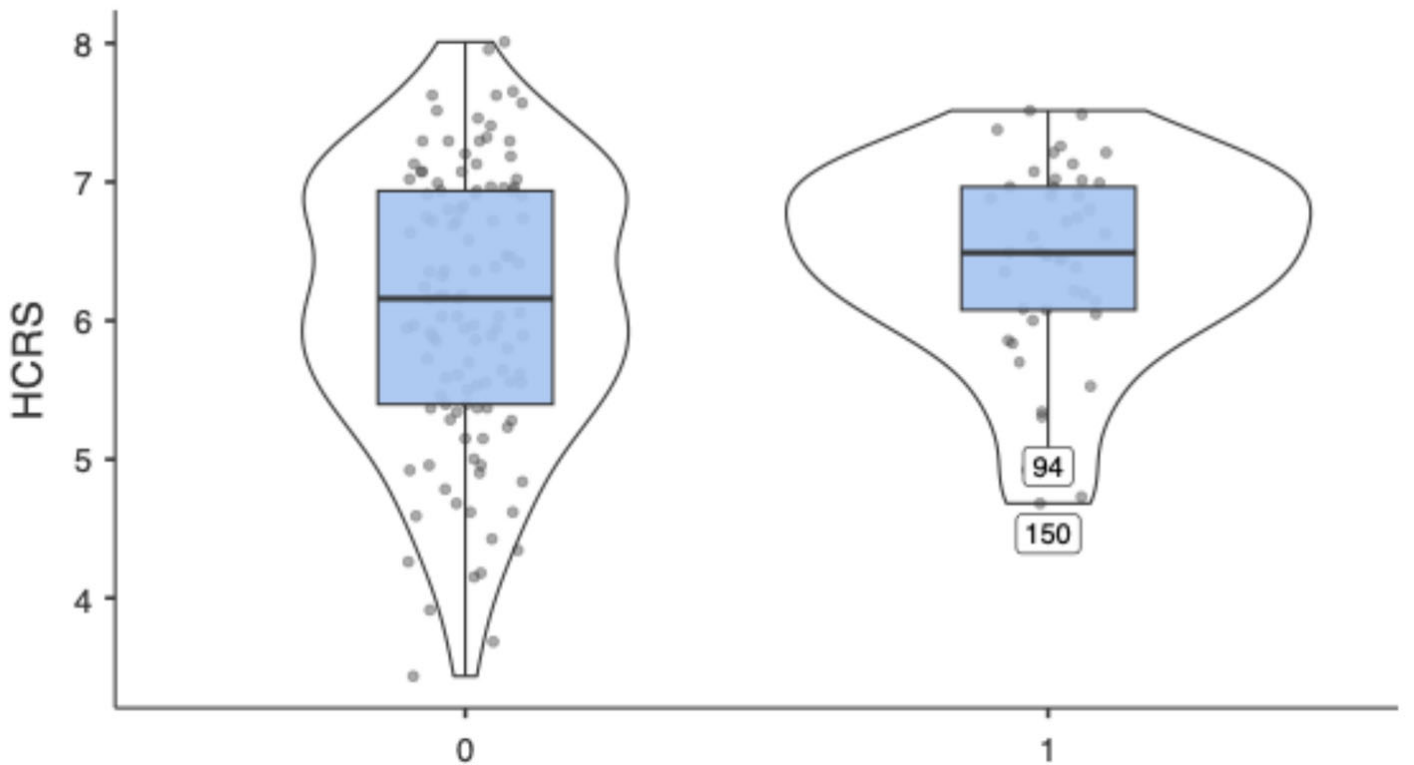
Boxplot of Haematuria Cancer Risk Score (HCRS) by tumour stage. Median HCRS was higher in MIBC than in non‐MIBC (6.49 vs. 6.16; *p* = 0.04). Group 1: non‐MIBC, group 2: MIBC.

The median HCRS was significantly higher in patients with MIBC than in those with non‐muscle‐invasive disease (6.49 [IQR 6.08–6.96] vs. 6.16 [IQR 5.44–6.94]; *p* = 0.04) (Figure 1).

This difference indicates that higher HCRS values tend to cluster among patients with muscle invasion.

### 3.3 Association between HCRS and MIBC

Univariate logistic regression demonstrated a significant association between HCRS and the presence of MIBC (OR = 1.78; 95% CI 1.03–3.06; *p* = 0.039). Because HCRS inherently integrates age, sex, haematuria type and smoking status, no multivariable model including these parameters was fitted to avoid collinearity. Exploratory analyses including CIS and variant histology yielded *p* = 0.14 and *p* = 0.28, respectively, indicating no additional predictive contribution.

Patients with HCRS ≥6 had significantly higher MIBC prevalence (36.4% vs. 14.3%, χ2 = 9.35, *p* = 0.002; Table 2). Univariate logistic regression confirmed a 3.4‐fold increased risk (OR 3.43, 95% CI 1.42–8.30, *p* = 0.003).

**TABLE 2:** Distribution of MIBC according to HCRS threshold. Patients with HCRS ≥6 had significantly higher MIBC prevalence (36.4% vs. 14.3%, χ^2^ = 9.35, *p* = 0.003).

HCRS group	Non‐MIBC *n*, %	MIBC *n*, %	Total, *n*	OR (95% CI)	*p* value
Low (<6) *n*, %	54 (85.7%)	9 (14.3%)	63		
High (≥6) *n*, %	63 (63.6%)	36 (36.4%)	99	3.43 (1.42–8.30)	**0.003**
Total *n*, %	117 (72.2%)	45 (27.8%)	162		

### 3.4 Discriminative performance

ROC analysis, using HCRS as a continuous variable, revealed limited discriminative ability for predicting muscle invasion (AUC = 0.60; 95% CI 0.53–0.67). The Youden‐derived optimal cut‐off was 6.04, with sensitivity 77.8%, specificity 48.3%, PPV 38.6% and NPV 84.1%. For clinical interpretability, an integer threshold of HCRS ≥6 provided near‐identical performance, supporting the practical use of rounded values in routine triage settings. Given the relatively low prevalence of MIBC (27.8%), the low PPV and high NPV were expected. Low PPV reflects the low prevalence of MIBC in the cohort, underscoring that the HCRS performs better as a screening‐oriented rather than exclusionary tool for invasive disease.

## 4 DISCUSSION

This study examined the prognostic relevance of the HCRS in predicting MIBC among patients presenting with haematuria.

In a cohort of 162 patients, higher HCRS values were significantly associated with muscle invasion; however, the overall discriminative ability of the score was limited (AUC = 0.60; 95% CI 0.53–0.67). At the Youden‐derived threshold of 6.04, sensitivity approached 78%, but specificity remained modest (48%). These findings indicate that while elevated HCRS values tend to accompany more aggressive disease, the score alone has suboptimal accuracy for differentiating muscle‐invasive from non‐muscle‐invasive tumours.

Our results conceptually extend the diagnostic foundation of the original HCRS, which was developed by Tan et al. to stratify cancer risk among patients with hematuria.^6^, ^7^ Although the original model demonstrated good to excellent diagnostic accuracy (AUC 0.82–0.86) and has been externally validated, its ability to predict tumour aggressiveness or stage progression has remained uncertain. The modest prognostic signal observed in the present study is biologically plausible, as the individual components of the HCRS—visible haematuria, older age, male sex and smoking—are themselves established risk factors for high‐grade and advanced‐stage disease.^10^, ^11^ The biological rationale for this association is supported by prior literature: Visible haematuria correlates with larger and higher‐grade tumours, smoking is known to promote carcinogenesis and invasion, and male sex and advanced age are associated with more aggressive phenotypes.^12^, ^13^, ^14^

Although the CI for the odds ratio was relatively wide (1.42–8.30), this is expected given the modest sample size and low event rate in the HCRS <6 group. The statistically significant *p* value (0.003) and consistent direction of effect across univariate and continuous analyses support the robustness of the finding. Future studies with larger cohorts are needed to narrow the CI and confirm the magnitude of risk.

The attenuation of predictive strength (AUC = 0.60) likely reflects both collinearity among the HCRS components and the multifactorial nature of tumour invasion. Because the score inherently incorporates age, sex and haematuria type, multivariable adjustment including these parameters was not performed to prevent redundancy. Instead, the study focused on the score's overall discriminative capacity as a single, clinically usable index. This approach allows a fair evaluation of the HCRS as it would be applied in real‐world triage settings, where individual variables are not re‐entered into predictive equations.

From a clinical perspective, the current findings suggest that the HCRS—though not independently sufficient to predict muscle invasion—may still provide complementary information for patient stratification. Patients with higher HCRS values may merit closer diagnostic and oncologic surveillance, particularly when combined with other adverse features such as carcinoma in situ (CIS), variant histology or imaging findings suggestive of deep invasion. Practically, an HCRS ≥6 may prompt expedited cystoscopy or enhanced imaging (e.g. multiparametric MRI), while lower scores could support deferred invasive evaluation in select low‐risk patients.

Heard et al. recently combined the HCRS with the Oncuria‐Detect urine biomarker test, achieving an AUC of 0.91 and a negative predictive value of 0.97 for bladder cancer detection.^15^ Their results underscore the strong *diagnostic* potential of HCRS, particularly when integrated with molecular assays. In contrast, our findings highlight its prognostic relevance for muscle invasion, suggesting that simple clinicodemographic parameters may still offer meaningful insight into tumour aggressiveness and could complement emerging biomarker‐based models.

This study has several limitations. Its retrospective, single‐centre design and modest sample size may limit generalizability. The exclusion of patients with missing data could introduce selection bias, although complete‐case analysis ensured data consistency.

Furthermore, muscle invasion is influenced by numerous biological and molecular determinants not captured by clinical scores such as the HCRS. Nevertheless, the present analysis represents one of the first attempts to evaluate the prognostic dimension of the HCRS in a pathologically confirmed cohort using a transparent, reproducible R‐based workflow.

## 5 CONCLUSION

The HCRS showed a modest association with MIBC but only limited discriminative performance (AUC = 0.60).

While primarily designed as a diagnostic triage tool, higher HCRS values were more common among patients with muscle‐invasive disease. An HCRS threshold of ≥6 provided clinically interpretable performance comparable to the statistical optimum and may help identify patients warranting closer evaluation. The HCRS may therefore support risk stratification in haematuria pathways by flagging individuals at higher risk of muscle invasion—particularly when combined with cystoscopy and imaging findings.

## AUTHOR CONTRIBUTIONS

**Serdar Turan:** Study concept and design; data acquisition; analysis and interpretation of data; drafting of the manuscript; critical revision of the manuscript. **Rifat Burak Ergul:** Statistical analysis and interpretation of data. **Cem Tugrul Gezmis, Nusret Can Cilesiz, Mehmet Uzut, Mustafa Satılmısoglu,** and **Mustafa Bahadır Can Balcı:** Data acquisition; critical revision of the manuscript for important intellectual content. All authors read and approved the final manuscript.

## CONFLICT OF INTEREST STATEMENT

The authors declare no conflicts of interest.

## ETHICS STATEMENT

This study was approved by the Institutional Ethics Committee of Taksim Training and Research Hospital (approval no.: 50; date: 09‐07‐2025). The requirement for informed consent was waived due to the retrospective nature of the study.
